# Selective HDAC6 inhibition protects against blood–brain barrier dysfunction after intracerebral hemorrhage

**DOI:** 10.1111/cns.14429

**Published:** 2023-09-04

**Authors:** Cuiying Peng, Yilin Wang, Zhiping Hu, Chunli Chen

**Affiliations:** ^1^ Department of Neurology, Second Xiangya Hospital Central South University Changsha Hunan China; ^2^ Department of Neurology, Hunan Provincial Rehabilitation Hospital Hunan University of Medicine Changsha Hunan China

**Keywords:** blood–brain barrier, HDAC6, histone deacetylase inhibitors, intracerebral hemorrhage, tubastatin A

## Abstract

**Backgrounds:**

Blood–brain barrier (BBB) disruption after intracerebral hemorrhage (ICH) significantly induces neurological impairment. Previous studies showed that HDAC6 knockdown or TubA can protect the TNF‐induced endothelial dysfunction. However, the role of HDAC6 inhibition on ICH‐induced BBB disruption remains unknown.

**Methods:**

Hemin‐induced human brain microvascular endothelial cells (HBMECs) and collagenase‐induced rats were employed to investigated the underlying impact of the HDAC6 inhibition in BBB lesion and neuronal dysfunction after ICH.

**Results:**

We found a significant decrease in acetylated α‐tubulin during early phase of ICH. Both 25 or 40 mg/kg of TubA could relieve neurological deficits, perihematomal cell apoptosis, and ipsilateral brain edema in ICH animal model. TubA or specific siRNA of HDAC6 inhibited apoptosis and reduced the endothelial permeability of HBMECs. HDAC6 inhibition rescued the degradation of TJ proteins and repaired TJs collapses after ICH induction. Finally, the results suggested that the protective effects on BBB after ICH induction were exerted via upregulating the acetylated α‐tubulin and reducing stress fiber formation.

**Conclusions:**

Inhibition of HDAC6 expression showed beneficial effects against BBB disruption after experimental ICH, which suggested that HDAC6 could be a novel and promising target for ICH treatment.

## Graphical abstract

The expression of acetylated α‐tubulin decreased in the early stage of intracerebral hemorrhage. HDAC6 inhibition by TubA or siRNA treatment rescued the degradation of TJ proteins and decreased BBB permeability via inhibiting actin stress fiber formation after ICH.

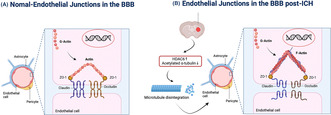

## 1 INTRODUCTION

Intracerebral hemorrhage (ICH) is a common clinically acute cerebrovascular disease characterized by primary non‐traumatic hemorrhage in the brain parenchyma.^1^, ^2^ Despite recent advances in treating ICH, the clinical outcomes remain unsatisfactory with merely approximately one fifth of patients restoring functional independence after 6 months.^3^ Blood–brain barrier (BBB) destruction is highly regarded as a symbol of secondary brain injury induced by ICH. Besides, vasogenic brain edema caused by endothelial dysfunction is supposed to be a crucial contributor to poor long‐term prognosis.^4^, ^5^ Thus, it was vital to dig new therapies to combat the BBB damage.^6^

Histone deacetylase inhibitors (HDACis) have been found to act on stroke and neurodegenerative diseases via various mechanisms in recent years.^7^, ^8^, ^9^, ^10^ Pan‐ and isoform‐specific HDACis play roles in different cerebral diseases including hemorrhagic stroke.^11^ HDAC6 belongs to the class IIb HDACs, mainly locates in cytoplasm and possesses two catalytic domains.^12^ Tubastatin A (TubA) is the most potent inhibitor of HDAC6 and with over 1000‐fold selectivity to HDAC6.^13^ Notably, genetic or pharmacologic inhibition of HDAC6 has been found to exert neuroprotective effects in ischemia stroke,^14^, ^15^ Huntington's disease,^16^, ^17^ Alzheimer's Disease,^17^ and hemorrhagic stroke.^18^, ^19^ Besides, researchers found that HDAC6 knockdown by small interfering RNA or TubA can control the dynamics of endothelial barrier integrity in pulmonary edema models.^20^ As we know, endothelial barrier integrity is essential for maintaining the function of BBB. However, whether pharmacological inhibition or gene interference of HDAC6 improves BBB function after ICH remain unclear. Thus, our study designed to explore the protective roles of HDAC6 inhibition on BBB leakage in ICH model and its related mechanisms.

## 2 MATERIALS AND METHODS

### 2.1 Experimental design

Experiment I: Investigate the ICH‐induced effects on the expression of acetylated α‐tubulin. Rats were randomly distributed into six subgroups: sham group, ICH 6 h, ICH 1d, ICH 2d, ICH 3d, ICH 7 day. The ipsilateral hemisphere perihematoma tissue were collected for western blot analysis in each group (*n* = 3/group). Immunofluorescence staining was conducted in ICH 3d group (*n* = 3/group). HBMECs were arranged into six groups and treated with different concentrations of hemin for 24 and 48 h to observe cells viability. Hemin at 100 μM for 24 h was chosen to detect the protein level of acetylated α‐tubulin.

Experiment II: Investigate the neuroprotective effects of HDAC6 inhibition after ICH stimulation. Rats were randomly assigned to four groups: (1) sham; (2) ICH + vehicle; (3) ICH + TubA (25 mg/kg); (4) ICH + TubA (40 mg/kg). Neurological function (*n* = 6/group) was evaluated at 1 and 3 days post‐ICH. TUNEL staining was conducted (*n* = 3/group) at 3 days post‐ICH. HBMECs were sorted into 5 groups: (1) control group; (2) Hemin + vehicle group; (3) Hemin + TubA (3 μM); (4) Hemin + NC‐siRNA; (5) Hemin + HDAC6 siRNA. CCK8 and flow cytometry analysis were performed at 24 h after hemin‐induced in each group.

Experiment III: Investigate the effect of HDAC6 inhibition on BBB injury against ICH induction. The subgroups in vivo and in vitro were the same as experiment II. Brain water content assay (*n* = 5) and Evans blue (EB) extraction assay (*n* = 3) was performed to evaluate the leakage of BBB at 1 and 3 days post‐ICH. FITC‐dextran transwell analysis was applied to detect the endothelial cells permeability.

Experiment IV: Probe the potential molecular mechanism of HDAC6 inhibition on early brain injury post‐ICH. The subgroups were consistent with experiment II. Thirty‐six rats were sacrificed at 3 days post‐ICH. The TJ protein expressions (*n* = 3) were measured by Western blot, the ultrastructure of TJs (*n* = 3) was observed by TEM and F‐actin (*n* = 3) was evaluated by immunofluorescence assay in each group. The above tests were also performed in vitro.

### 2.2 Animals

Adult, male SD rats (275~325 g) were obtained from Hunan Slack Jingda Laboratory (Changsha, China) and housed in central laboratory of Hunan Provincial People's Hospital at a fixed temperature (25°C) and relative humidity (60%). All experimental protocols were approved by the Animal Care and ethics review committee of Central South University (IACUC approval No: 2020079). SD rats were fed with free water and food, dwelt in a 12 h light/dark cycle. Assignment and use of rats is clarified in Table S1.

### 2.3 Rat model of ICH

The ICH procedure was performed via injecting collagenase type IV (Sigma‐Aldrich) as previously published with minor modifications.^21^, ^22^ In simple terms, rats were deeply anesthetized with pentobarbital sodium (40 mg/kg) by intraperitoneal injection. Stereotactically insert a microsyringe (10 μL) into the right striatum across the cranial borehole. Collagenase IV (0.2 U) dissolved in 2 μL of 0.9% saline was injected gradually for 10 min. The sham operation was injected an equal volume of saline only.

### 2.4 Cell culture and model of ICH

HBMECs were cultured in Dulbecco's modified Eagle's medium (DMEM), with fetal bovine serum (10%, Gibco) and penicillin–streptomycin (1%, Gibco) at 37°C with 5% CO2. HBMECs were treated with hemin (100 uM, #C3984, ApexBio) for 24 h to induce an ICH model in vitro.

### 2.5 Drugs administration

For animals, TubA (#A4101, ApexBio) was injected intraperitoneally 30 min before ICH induction with two dosages: 25 and 40 mg/kg.^14^, ^17^ In vitro, HBMECs were pretreated with TubA (3 μM) dissolved in DMSO for 6 h as previously described.^20^

### 2.6 SiRNAs and in vitro transfection

The specific siRNAs against HDAC6 were purchased from HonorGene (siG160718025500, Changsha, CHN). We tested three different siRNAs interference efficiencies, and the most effective one was applied to the following research (Sequence: GAAACAACCCAGTACATGAAT) (Table S2). HDAC6 siRNA or control siRNA was transfected through Lipofectamine 3000 (Invitrogen, L3000‐015) for 48 h in accordance with the manufacturer's instructions.

### 2.7 Assessment of neurological function

The neurological function was assessed at 1 and 3 days post‐ICH by Garcia scoring system,^23^ which includes six individual tests: voluntary movement, symmetry of limbs, forelimbs extension, body proprioception, climbing ability, and tentacle touch. Each test scores from 0 to 3. All trials conduction, calculation, and evaluation were subjected by two independent trained investigators.

### 2.8 Blood–brain barrier permeability in vivo

We evaluated blood–brain barrier permeability by brain water content (BWC) measurement and Evans blue (EB) staining. The brain tissue was dissected into 5 sections: contralateral and ipsilateral basal ganglia, contralateral and ipsilateral cortex, and cerebellum. Samples were instantly weighed on a precision electronic autobalance for wet weight (WW), then dehydrated in an oven at 100°C to obtain dry weight (DW). The calculation formula is: BWC = [(WW − DW)/WW] × 100%.

EB dye (2% in PBS, 4 mL/kg, Sigma‐Aldrich) was injected into the femoral vein and circulated for 1 h. Then, animals were perfused transcardially with PBS, and perihematoma tissue were harvested and homogenized in 3 mL formamide (Macklin, F810079) before incubated for 72 h, and then centrifugation at 10,000 rpm for 25 min. The fluorescence intensity of diluted supernatant was measured at 610 nm using a microplate reader and quantified according to a standard curve.

### 2.9 Endothelial cell permeability in vitro

The transwell assay was employed to detect the permeability of endothelial cell monolayer to FITC‐dextran. Briefly, HBMECs were inoculated in the cell chambers. After endothelial cells formed a monolayer, Fluorescein isothiocyanate (FITC)‐dextran (Sigma, 46944) was diluted in medium to 10 μg/mL and incubated for 20 min. Fluorescence intensity at wavelengths of 485 and 520 nm was tested by fluorescence plate reader.

### 2.10 Western Blot analysis

Samples from ipsilateral hemisphere were homogenized and centrifuged, then separated by SDS‐poly‐acrylamide gel electrophoresis, and transferred to nitrocellulose membranes. Next, we blocked the membrane with defatted milk (5%) and incubated it with the following primary antibodies: mouse anti‐ace‐α‐tubulin (1:2000, #ab24610, Abcam), anti‐α‐tubulin (1:2000, #ab52866, Abcam), rabbit anti‐ZO‐1 (1:2000, #21773‐1‐AP, Proteintech), rabbit anti‐occludin (1:2000, #ab216327, Abcam), or mouse anti‐β‐actin antibody (1:5000, #66009‐1‐Ig, Proteintech). Appropriate secondary antibodies were chosen to incubate for 90 min. Finally, the data were processing through the Image J software.

### 2.11 Immunofluorescence staining

The sections were permeabilized three times with 0.2% Triton X‐100 and incubated in sequence with primary antibodies and appropriate conjugated secondary antibodies. All antibodies were same as used in WB assay. In addition, F‐Actin was stained with iFluor 647 (ab176759, 1:1000, Abcam) in brain paraffin sections and HBMECs. The images were viewed on a confocal microscope (Nikon, Nikon Eclipse C1).

### 2.12 TUNEL staining

TUNEL staining was administered to access the death of cells and performed following the manual of the Cell Apoptosis Detection Kit (G1502‐50 T, ServiceBio). The TUNEL‐positive cells were captured by the fluorescence microscope (Nikon Eclipse C1) and analyzed with Image J software.

### 2.13 Measurement of apoptosis in vitro

The flow cytometric analysis was employed to detect the apoptosis of HBMECs. In short, the HBMECs were collected by trypsin digestion and rinsed twice with PBS. The suspension was blended with Annexin V‐APC (5 μL) and then added PI (5 μL) to reincubated for 10 min. Finally, the mixture was instantly analyzed by a FACS flow cytometer C6 (FCM; FACSCanto II; BD Biosciences) and the results were analyzed by FlowJo v10.7.1 (Tree Star).

### 2.14 TEM analysis

We used transmission electron microscope (TEM) to observe the ultrastructure of TJs after ICH induction. All transmission electron microscopy was performed at Electron microscope Centre of Central South University. Brain tissues or HBMECs were prefixed with 2% glutaraldehyde in 0.1 M sodium phosphate buffer (PH 7.4) for 12 h at 4°C. After washing, samples were postfixed for 2 h with 1% osmium tetroxide and then gradually dehydrated in ascending concentrations of ethanol. The samples were then transferred to propylene oxide, embedded in Eponate 12 Resin and cut into 70 nm (Leica UC7). Subsequently, the sections were stained with uranyl acetate. Micrographs were acquired by a TEM (HT‐7700, Hitachi, Japan).

### 2.15 Statistical analysis

Normality was evaluated through the Shapiro–Wilk test, and all data were normally distributed. Comparison of multiple groups were analyzed by one‐way ANOVA followed by Bonferroni's post‐hoc tests. All values are presented as means ± SEM. *p* < 0.05 was considered statistically significant. Statistical analyses were performed via SPSS software.

## 3 RESULTS

### 3.1 Expression of acetylated α‐tubulin after ICH induction in vivo and in vitro

**FIGURE 1:**
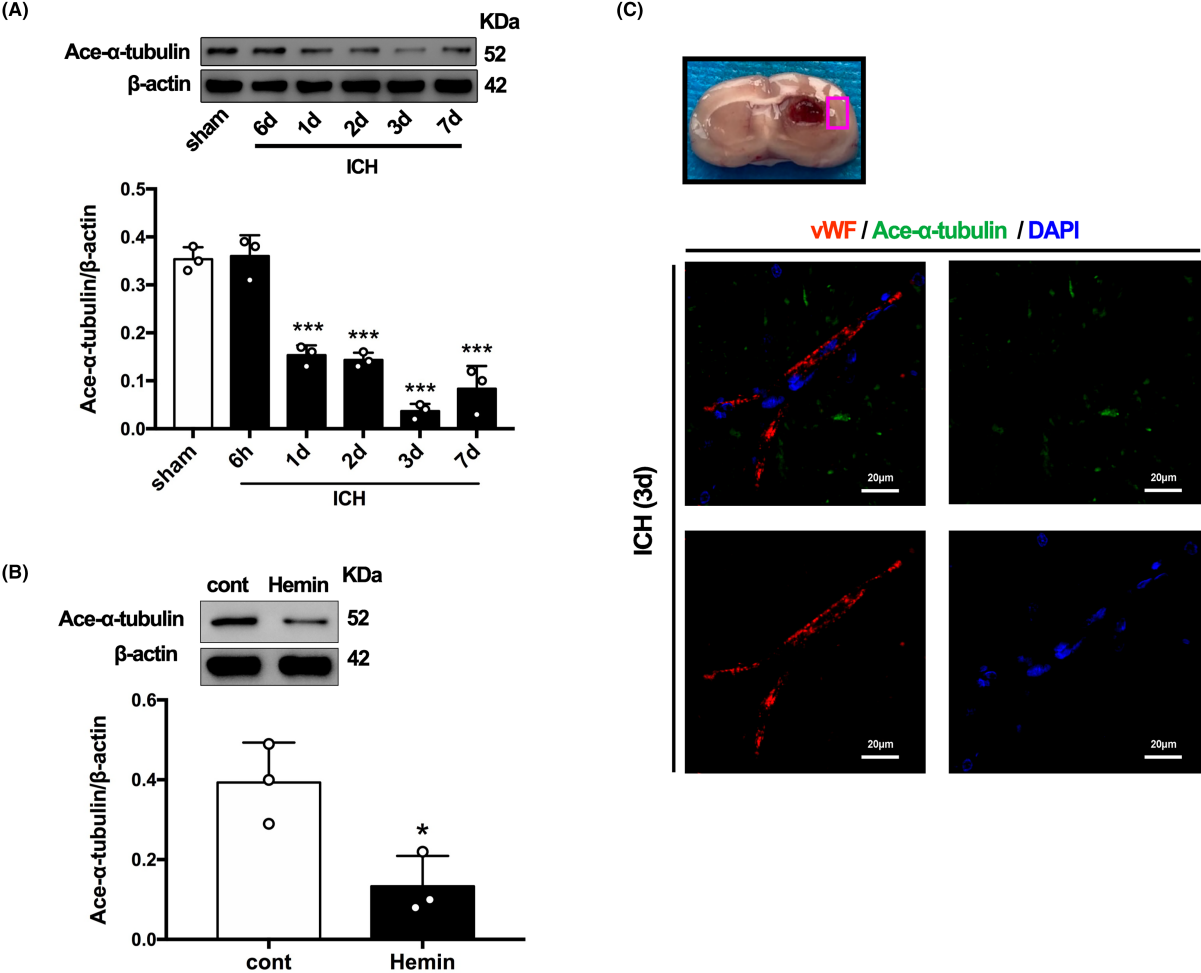
Time‐dependent trends of acetylation α‐tubulin protein levels in ICH rats and hemin‐induced HBMECs. (A) Western blot and quantification analysis of acetylated α‐tubulin in ICH animal model. Values are indicated by means ± SEM; versus sham, *p < 0.05; **p < 0.01; ***p < 0.001. (B) Western blot and quantification analysis of acetylated α‐tubulin in hemin‐induced HBMECs model. Values are indicated by means ± SEM; versus control, *p < 0.05; **p < 0.01; ***p < 0.001. (C) Double immunostaining of acetylated α‐tubulin with von Willebrand Factor (VWF). Scale bar = 20 μm.

The protein level of acetylated α‐tubulin from perihematoma tissue was monitored by WB analysis. Compared with the sham group, acetylated α‐tubulin decreased at 1 day post‐ICH, reached its lowest level at 3 days and then gradually rebounded at 7 days (Figure 1A). The expression of acetylated α‐tubulin in cultured HBMECs also decreased significantly after hemin treatment for 24 h (Figure 1B). Meanwhile, we observed the localization of acetylated α‐tubulin in the perihematoma tissue. The expression and distribution of acetylated α‐tubulin was further identified by immunohistochemical staining at 3 days post‐ICH. As shown in Figure 1C, we barely found acetylated‐α‐tubulin co‐localization with endothelial cells of rat brain at 3 days post‐ICH.

### 3.2 Neuroprotection effects of HDAC6 inhibition in vivo and in vitro

**FIGURE 2:**
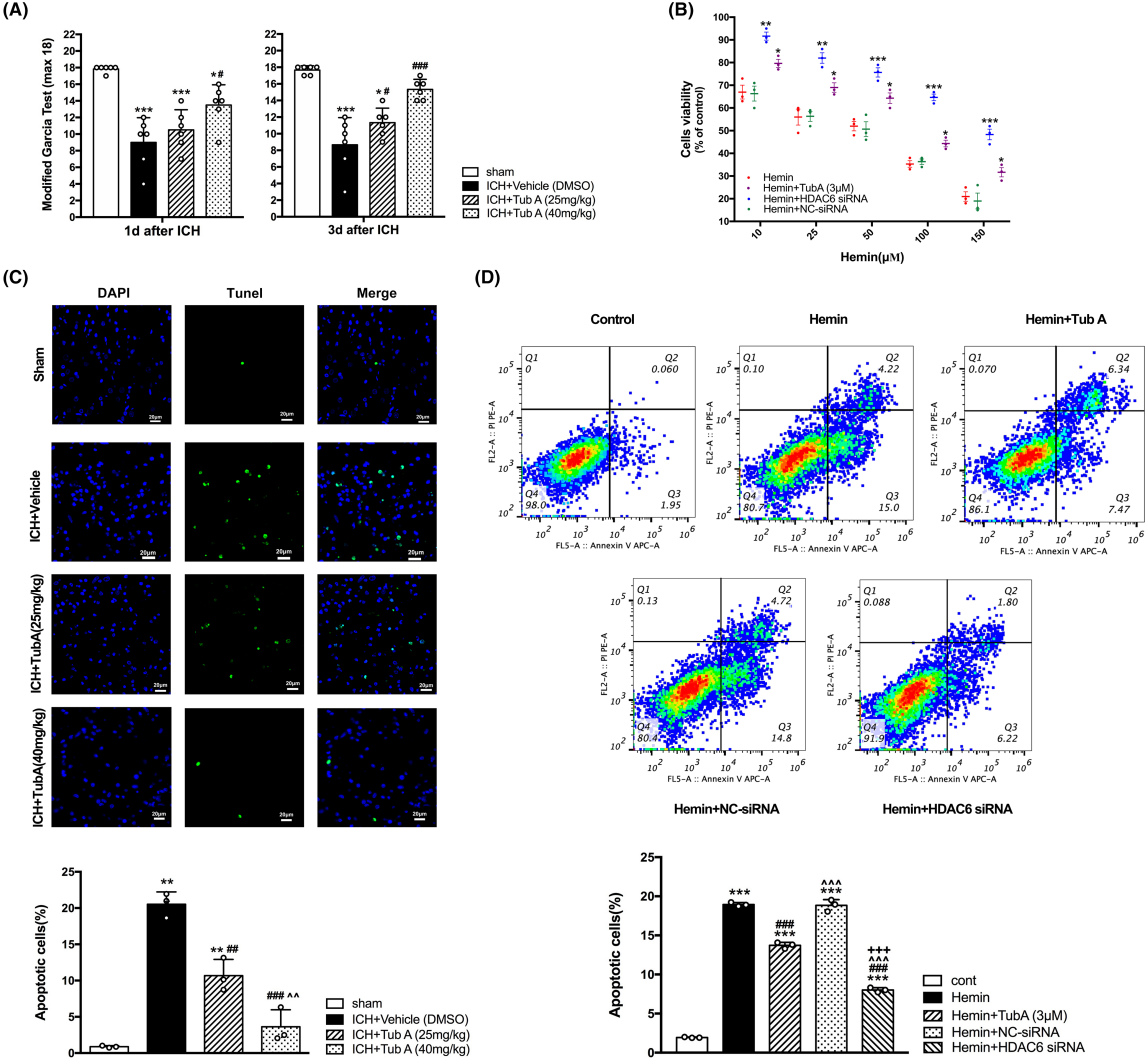
Effects of HDAC6 inhibition on ICH‐induced cerebral impairments. (A) Neural function valued by modified Garcia score test in ICH animal model. versus sham, *p < 0.05; **p < 0.01; ***p < 0.001. versus ICH + Vehicle, #*p* < 0.05; ##*p* < 0.01; ###*p* < 0.001. versus ICH + TubA (25 mg/kg), ^*p* < 0.05; ^^*p* < 0.01; ^^^*p* < 0.001. (B) Cellular viability assessed by CCK8 assay in hemin‐induced HBMECs model. versus hemin, *p < 0.05; **p < 0.01; *** *p* < 0.001. (C) Representative photographs and Quantitative analysis of TUNEL‐positive cells in rats. Scale bar =20 μm. versus sham, *p < 0.05; **p < 0.01; ***p < 0.001. versus ICH + Vehicle, #*p* < 0.05; ##*p* < 0.01; ###*p* < 0.001. versus ICH + TubA (25 mg/kg), ^*p* < 0.05; ^^*p* < 0.01; ^^^*p* < 0.001. (D) Cell apoptosis assessed by cytometric analyses in hemin‐induced HBMECs model. versus cont, *p < 0.05; **p < 0.01; ***p < 0.001. versus Hemin, #*p* < 0.05; ##*p* < 0.01; ###*p* < 0.001. versus Hemin + TubA (3 μM), ^*p* < 0.05; ^^*p* < 0.01; ^^^*p* < 0.001. versus Hemin + NC‐siRNA, +*p* < 0.05; ++*p* < 0.01; +++*p* < 0.001. Values are indicated by means ± SEM.

The ICH‐induced rats displayed severe neurological impairments at 1 and 3 days post‐ICH by modified Garcia test (vehicle vs. sham, *p* < 0.001, Figure 2A). Administration of TubA with high dose (40 mg/kg) were significant effective for the improvement of neurological impairments from day 1 to day 3 post‐ICH, while medium dose TubA (25 mg/kg) could improve neurological deficits only on day 3. Consistent with the behavioral results, the quantity of TUNEL‐positive cells in TubA‐treated group were obviously reduced compared with ICH group (vehicle vs. TubA 25 mg/kg, *p* = 0.001, vehicle vs. TubA 40 mg/kg, *p* < 0.001, Figure 2C). In vitro, CCK‐8 was conducted to assess the effect of HDAC6 inhibition on HBMECs viability. As expected, hemin‐induced cells viability was significantly improved while treated with TubA or specific HDAC6 knockdown by siRNA transfection (Figure 2B). Correspondingly, the apoptosis rate of hemin‐induced HBMECs was ameliorated significantly by siRNA treatment or TubA treatment (*p* < 0.001, Figure 2D).

### 3.3 HDAC6 inhibition attenuated ICH‐induced BBB disruption in vivo and in vitro

**FIGURE 3:**
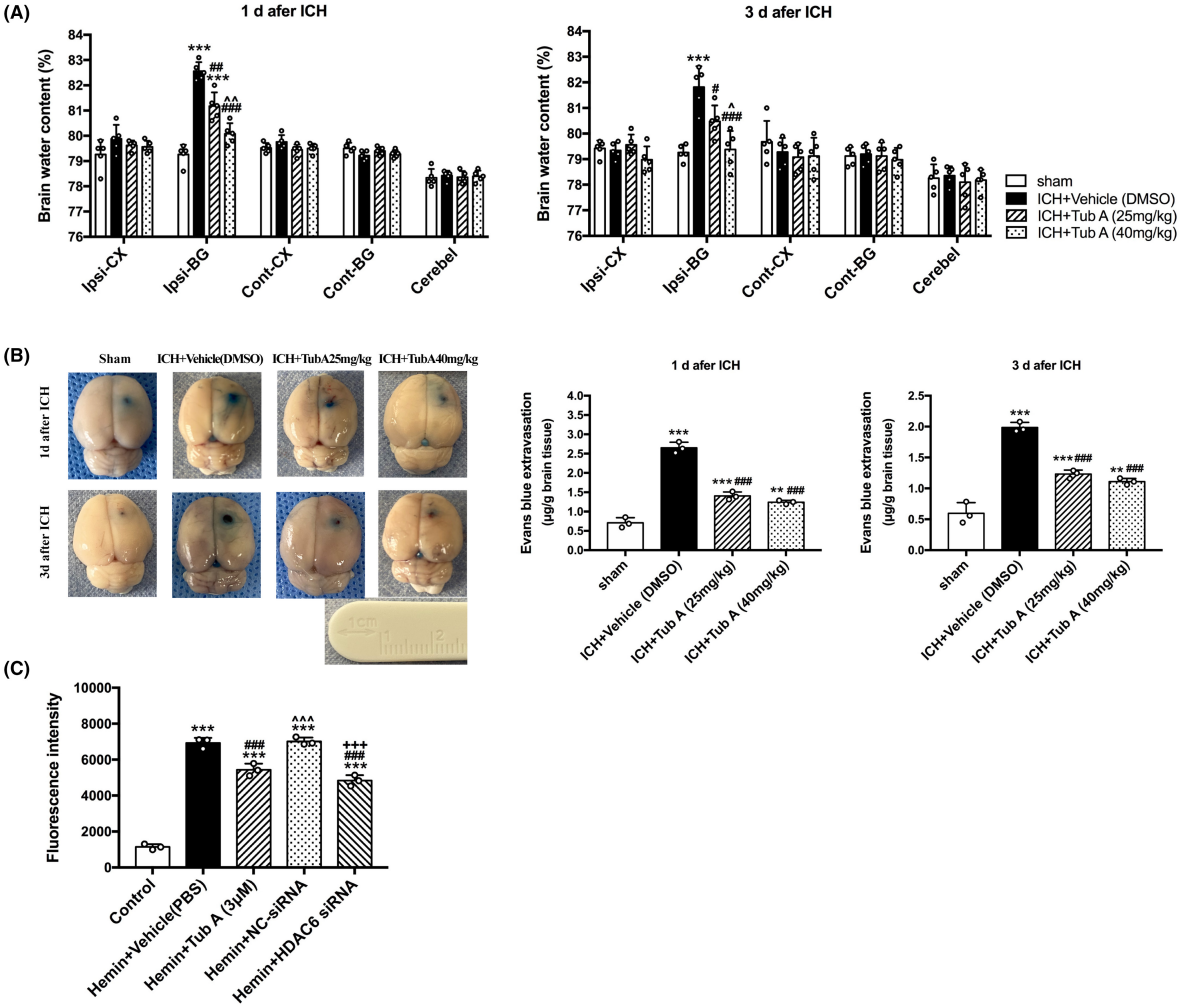
Effects of HDAC6 inhibition on ICH‐induced brain edema and BBB disruption. (A) Brain water content assessed by dry‐wet weight way in rats. Cont: contralateral; Ip: ipsilateral; BG: basal ganglia; CX: Cortex; Cerebel: cerebellum. versus sham, *p < 0.05; **p < 0.01; ***p < 0.001. versus ICH + Vehicle, #*p* < 0.05; ##*p* < 0.01; ###*p* < 0.001. versus ICH + TubA (25 mg/kg), ^*p* < 0.05; ^^*p* < 0.01; ^^^*p* < 0.001. (B) Quantitative analysis of Evans blue extravasation in rats. versus sham, *p < 0.05; **p < 0.01; ***p < 0.001. versus ICH + Vehicle, #*p* < 0.05; ##*p* < 0.01; ###*p* < 0.001. versus ICH + TubA (25 mg/kg), ^*p* < 0.05; ^^*p* < 0.01; ^^^*p* < 0.001. (C) Hemin‐induced endothelial permeability to FITC‐dextran measured in HBMECs. versus cont, *p < 0.05; **p < 0.01; *** *p* < 0.001. versus Hemin, #*p* < 0.05; ##*p* < 0.01; ###*p* < 0.001. versus Hemin + TubA (3 μM), ^*p* < 0.05; ^^*p* < 0.01; ^^^*p* < 0.001. versus Hemin + NC‐siRNA, +*p* < 0.05; ++*p* < 0.01; +++*p* < 0.001. Values are expressed as mean ± SEM.

After induction of ICH at 1 and 3 days, the BWC in ipsilateral basal ganglia area was significantly higher than sham group (*p* < 0.001, Figure 3A). Furthermore, TubA treatment did effectively reduce brain edema at doses of 25 mg/kg (vs. Vehicle, *p* = 0.001, 1 day; *p* = 0.024, 3 days) and 40 mg/kg (vs. Vehicle, *p* < 0.001, 1 and 3 days). Moreover, the effect of high‐doses group on brain edema is better than low‐doses group (TubA 40 mg/kg vs. TubA 25 mg/kg, *p* = 0.006, 1 day, *p* = 0.036, 3 days).

The Evans blue analysis showed obvious disruption of BBB both at 1 and 3 days after ICH induction. Moreover, the two doses of TubA we used markedly alleviated the EB dye extravasation in the ipsilateral hemisphere (vs. vehicle, *p* < 0.001, 1 and 3 days, Figure 3B), but there was no significant difference between them.

For in vitro experiment, we assessed the effects of HDAC6 inhibition on endothelial permeability after hemin induction. Hemin led to high permeability of HBMECs to FITC‐dextran, suggesting endothelial dysfunction. On contrast, TubA treatment and specific siRNA treatment significantly reduced the hemin‐induced endothelial permeability (*p* < 0.001, Figure 3C).

### 3.4 HDAC6 inhibition attenuates the degradation of TJ proteins and the disruption of TJ ultrastructure in vivo and in vitro

**FIGURE 4:**
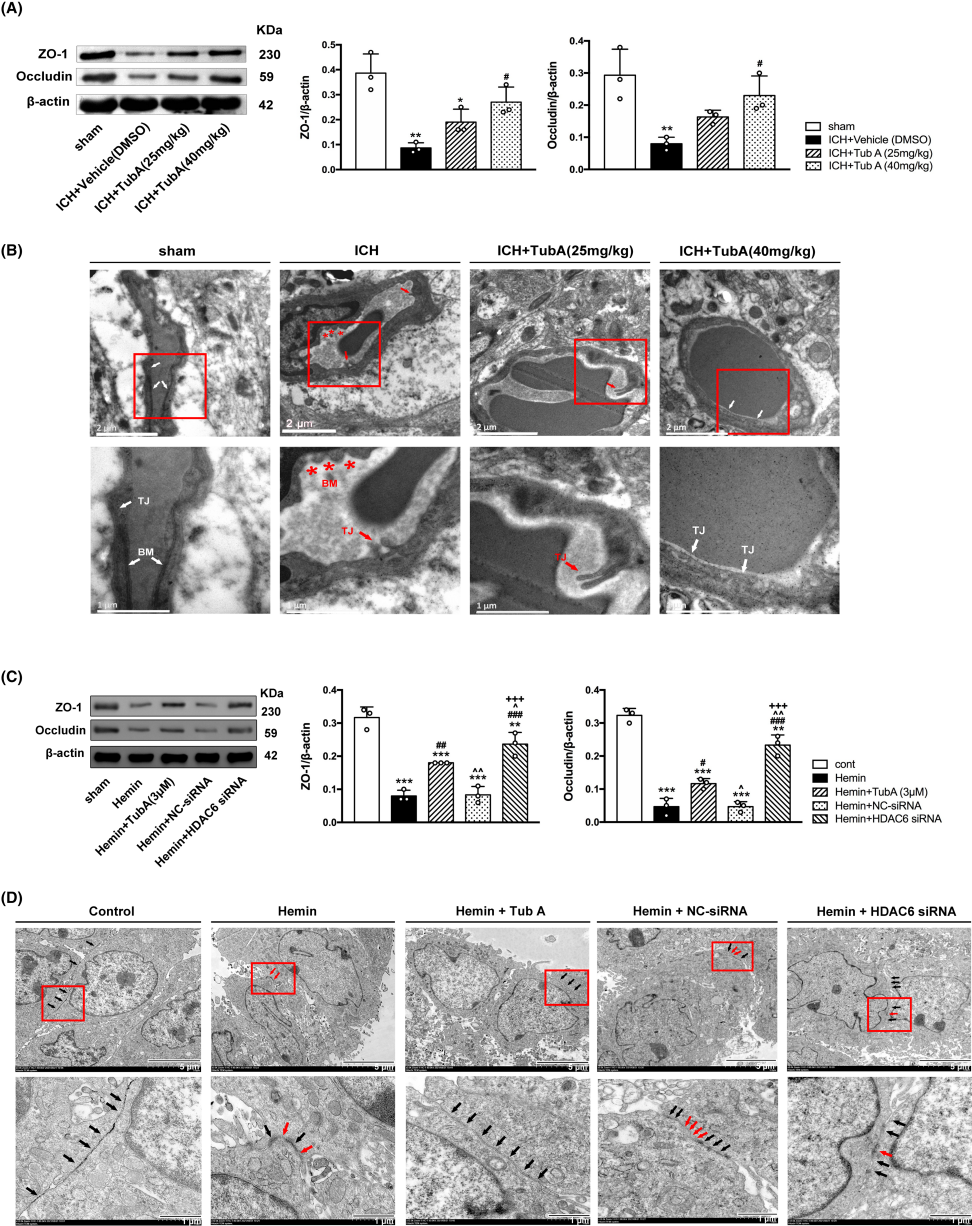
Effects of HDAC6 inhibition on tight junction in ICH rats and hemin‐induced HBMECs. (A) Western blot and quantification analysis of ZO‐1 and occludin in rats. versus sham, *p < 0.05; **p < 0.01; ***p < 0.001. versus ICH + Vehicle, #*p* < 0.05; ##*p* < 0.01; ###*p* < 0.001. versus ICH + TubA (25 mg/kg), ^*p* < 0.05; ^^*p* < 0.01; ^^^*p* < 0.001. (B) Representative electron microscopic images of TJ ultrastructure in rats. The red arrow indicates the broken TJs. The red asterisk represents desultory basal membrane. BM basal membrane, TJ tight junction. Scale bar = 2 and 1 μm. (C) Western blot and quantification analysis of ZO‐1 and occludin in HBMECs. versus cont, *p < 0.05; **p < 0.01; ***p < 0.001. versus Hemin, #*p* < 0.05; ##*p* < 0.01; ###*p* < 0.001. versus Hemin + TubA (3 μM), ^*p* < 0.05; ^^*p* < 0.01; ^^^*p* < 0.001. versus Hemin + NC‐siRNA, +*p* < 0.05; ++*p* < 0.01; +++*p* < 0.001. (D) Representative electron microscopic images of TJ ultrastructure in HBMECs. The black arrow indicates endothelial TJs. The red arrow indicates break down of TJs. Scale bar = 5 and 1 μm.

Here, we explored the influences of TubA on the expression of two essential TJ proteins (occludin and ZO‐1) after ICH induction. Our data indicated that ICH induced notable degradation of occludin and ZO‐1, while 40 mg/kg of TubA significantly increased these two TJ proteins (ZO‐1: Vehicle vs. TubA 40 mg/kg: *p* = 0.024; Occludin: Vehicle vs. TubA 40 mg/kg: *p* = 0.049, Figure 4A). For in vitro experiment, the level of occludin and ZO‐1 was significantly up‐regulated by TubA treatment in hemin‐induced HBMECs (Figure 4C). Consistently, our data also confirmed that specific HDAC6 knockdown by siRNA transfection exerted rescue effects on the degradation of ZO‐1 and occludin significantly (*p* < 0.05).

Meanwhile, ultrastructural changes of TJs in endothelial cells after ICH induction were primarily detected by TEM. Under normal conditions, it was found that the basal layer was integrate and continuous; the endothelial TJs appeared intact and contiguous. However, BBB ultrastructure was dramatically damaged at 3 day post‐ICH. As shown in Figure 4B, some TJs of endothelial cells were severely opened and seemed shorter and blurred. Meanwhile, the basement membrane appeared intermittent, irregular and thinner than sham group. However, TubA treatment (40 mg/kg) group showed the basal membrane of endothelial cells was more regular, smooth, and integrate, and the TJs were nearly intact (Figure 4B). Although there are breaks between the TJs in low dosage (25 mg/kg) group, they are longer and more intact than the Vehicle group.

In consistent with the in vivo study, the ultrastructural changes in HBMECs after HDAC6 inhibition were also detected. Compared with the hemin‐induced group, basal membranes of endothelial cells in HDAC6 siRNA group and TubA group were found to be more continuous and smoother. Moreover, TJs was longer and appeared as higher electronic density between adjacent cells (Figure 4D).

### 3.5 HDAC6 inhibition increased the expression of acetylated α‐tubulin and prevented subsequent actin stress fiber formation

**FIGURE 5:**
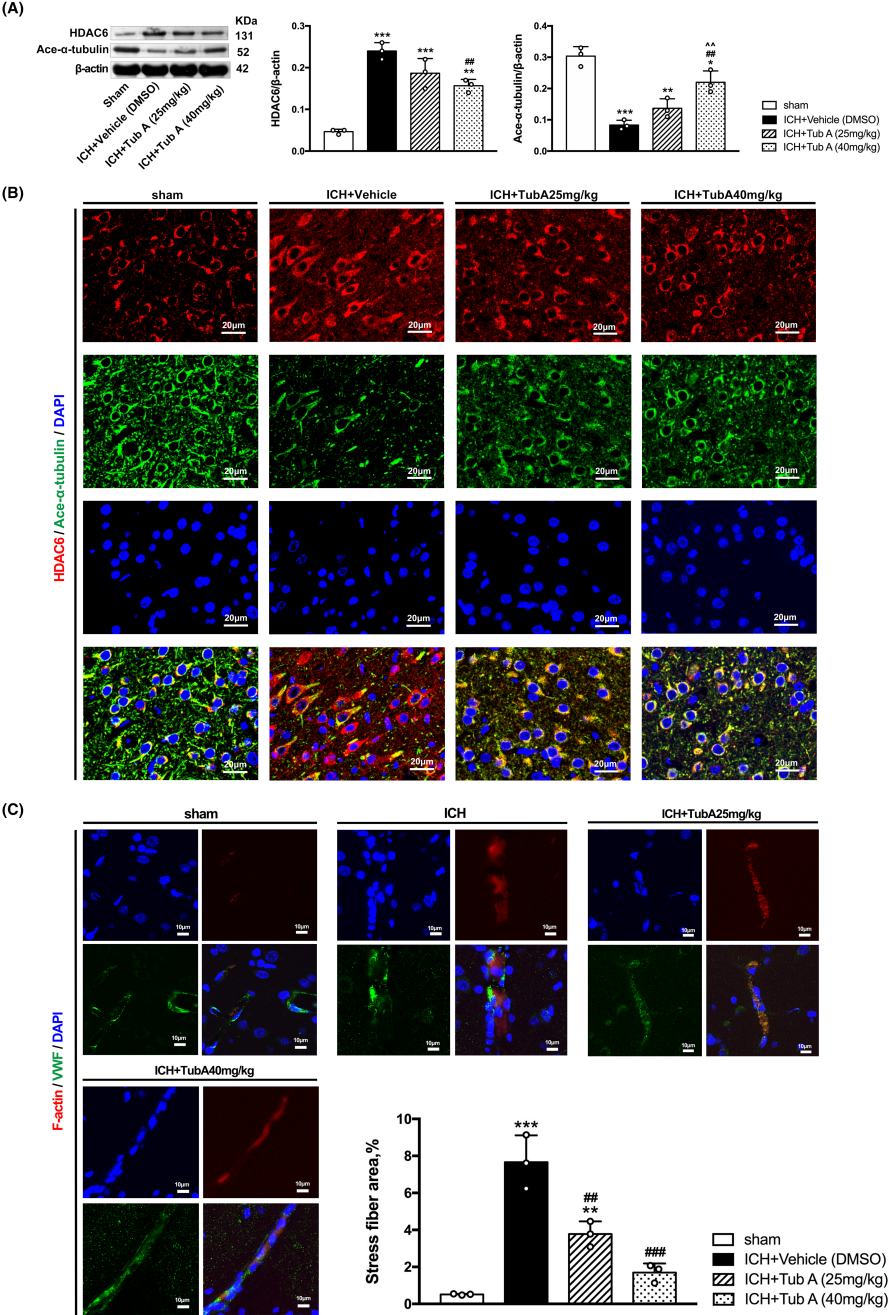
Inhibition of HDAC6 suppresses ICH‐induced α‐tubulin deacetylation and actin stress fiber formation in rats. (A) Western blot and quantification analysis of HDAC6 and acetylated α‐tubulin. (B) Double immunostaining of HDAC6 with acetylated α‐tubulin. Scale bar = 20 μm. (C) Double immunofluorescence staining for F‐actin and vWF. Scale bar = 10 μm. Values are expressed as mean ± SEM. versus sham, *p < 0.05; **p < 0.01; ***p < 0.001. versus ICH + Vehicle, #*p* < 0.05; ##*p* < 0.01; ###*p* < 0.001. versus ICH + TubA (25 mg/kg), ^*p* < 0.05; ^^*p* < 0.01; ^^^*p* < 0.001.

**FIGURE 6:**
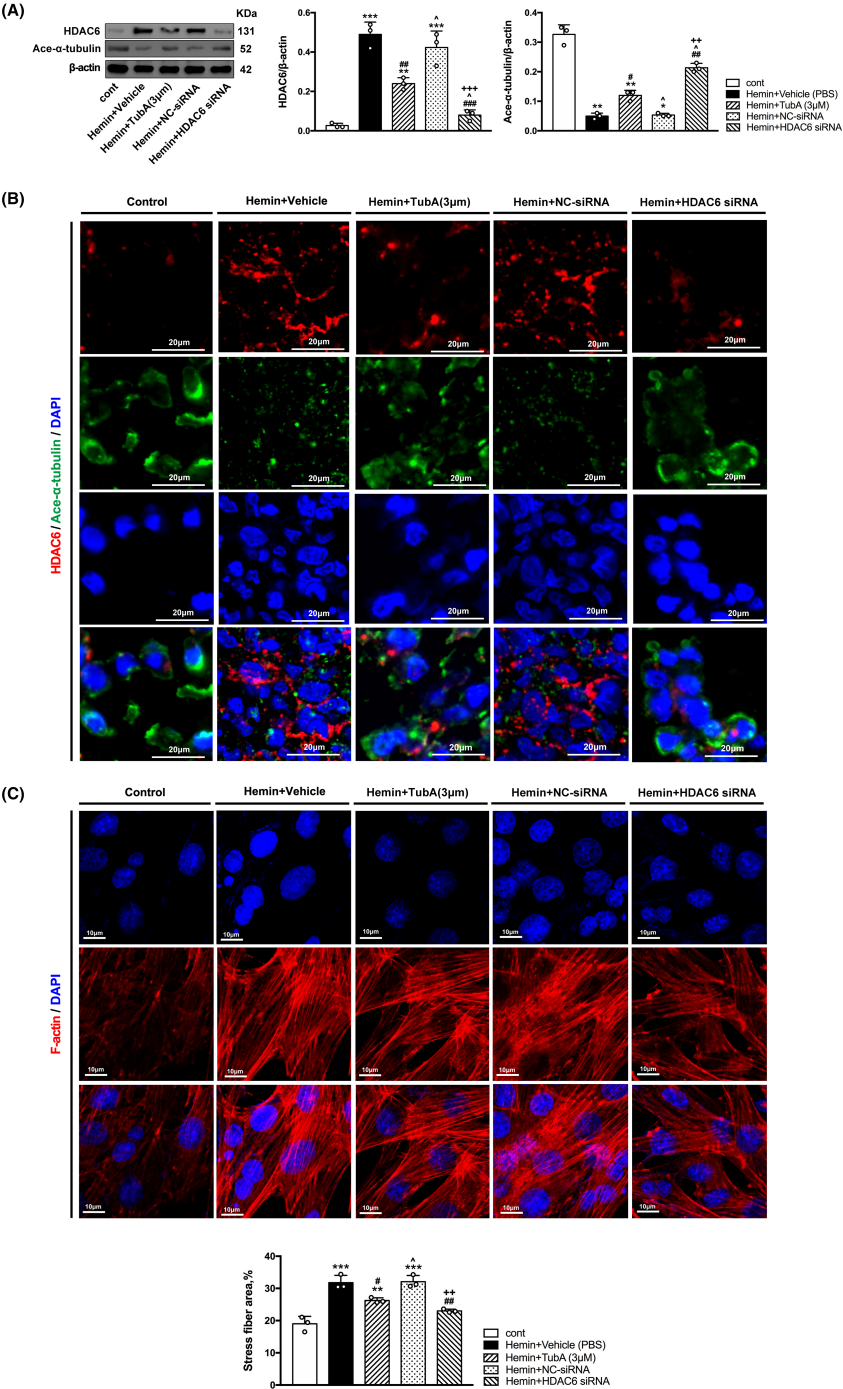
Inhibition of HDAC6 suppresses hemin‐induced α‐tubulin deacetylation and actin stress fiber formation in HBMECs. (A) Western blot and quantification analysis of HDAC6 and acetylated α‐tubulin. (B) Double immunostaining of HDAC6 with acetylated α‐tubulin. Scale bar = 20 μm. (C) Immunofluorescence staining for F‐actin. Scale bar = 10 μm. Values are expressed as mean ± SEM. versus cont, *p < 0.05; **p < 0.01; ***p < 0.001. versus Hemin, #*p* < 0.05; ##*p* < 0.01; ###*p* < 0.001. versus Hemin + TubA (3 μM), ^*p* < 0.05; ^^*p* < 0.01; ^^^*p* < 0.001. versus Hemin + NC‐siRNA, +*p* < 0.05; ++*p* < 0.01; +++*p* < 0.001.

For in vivo experiment, medium dosage of TubA (25 mg/kg) showed only a tendency to rescue the reduction of acetylated α‐tubulin (*p* = 0.322), while the high dosage of TubA (40 mg/kg) upregulated α‐tubulin acetylation after ICH induction significantly (vs. Vehicle, *p* = 0.003, Figure 5A). For in vitro experiment, the results confirmed that TubA treatment and HDAC6 knockdown both upregulated the expression of acetylated α‐tubulin in hemin‐induced HBMECs (Figure 6A). Besides, immunofluorescence staining was also performed for each group, and the results were consistent with western blot (Figures 5B and 6B).

Immunostaining results exhibited a distinct increase of phalloidin‐positive cells after ICH establishment in vivo and in vitro, which indicated actin stress fiber formation. Moreover, treatment with 25 and 40 mg/kg dosage of TubA both inhibited actin stress fiber formation (Figure 5C). The results of in vitro experiments still confirmed that TubA treatment and siRNA treatment inhibited actin stress fiber formation in hemin‐induced HBMECs (Figure 6C).

## 4 DISCUSSION

In the current job, we explored the role of HDAC6 inhibition and its underlying molecular mechanisms in early brain injury after ICH. The major observations are as follows: (1) The expression of acetylated α‐tubulin decreased in the early stage of intracerebral hemorrhage. (2) HDAC6 inhibition by TubA or siRNA treatment inhibited cell apoptosis after ICH. (3) HDAC6 inhibition by TubA or siRNA treatment rescued the degradation of TJ proteins (ZO‐1 and occludin) and reduced BBB permeability in ICH animal as well as cellular model. (4) TubA treatment increased α‐tubulin acetylation and inhibited actin stress fiber formation after ICH in ICH animal as well as cellular model. In general, these findings indicate that HDAC6 inhibition might be a promising treatment for protecting the BBB after ICH.

Histone deacetylases (HDACs) can catalyze the hydrolysis of acetyl groups from the lysine residues of histones and non‐histones, and play roles in epigenetics and signal modification.^24^ Accumulating evidences show that HDAC6‐targeted treatment is a considerable therapeutic strategy in central nervous system diseases for its neuroprotective and regenerative effects. On one hand, the ubiquitin‐binding‐ZnF of HDAC6 is involved in clearing the cytotoxic aggregates of misfolded proteins.^25^ On the other hand, high‐selective inhibitors or small interfering RNAs targeting HDAC6 exhibited neuroprotective effects in some neurological disease models. For instance, TubA and the shRNA of HDAC6 can ameliorate neuronal necroptosis from oxygen–glucose deprivation/reperfusion (OGDR),^15^, ^25^ alleviate cognitive dysfunction in mice with Alzheimer's disease (AD),^17^ and prevent axonal loss in mice with Charcot–Marie‐Tooth disease.^26^ Although the neuroprotective effects of HDAC6 inhibition have been extensively studied for many years, it was unclear whether HDAC6 inhibition play a role in ICH.

As the main substrate of HDAC6, acetylated α‐tubulin indirect represented the activity of HDAC6.^15^ Plenty of researches have studied the acetylated α‐tubulin levels in different neurological diseases. For instance, Wang et al. found remarkable reductions of acetylated α‐tubulin in the striatum and cortex at 24 and 72 h post‐ischemia.^14^ Besides, Zhang et al. observed acetylated α‐tubulin was dramatically decreased in transgenic mice model of AD.^17^ Moreover, Zeng et al. observed that α‐tubulin acetylation was significantly downregulated after OGDR in N2a cells.^15^ Recently, Yang et al. found both Dopamine (DA) neuron numbers and acetylated α‐tubulin levels decreased significantly at 7–28 days after ICH, and epothilone B (EpoB, a MT‐stabilizing agent) could ameliorated DA neuronal damage.^27^, ^28^ In addition, their team also demonstrated that promoting acetylation of α‐tubulin by TubA significantly alleviated axonal injury and mitochondrial dysfunction after ICH.^29^ In similarity with their studies, we observed a significant decline in acetylated α‐tubulin at 1 day after ICH, suggesting that the down‐regulation of acetylated α‐tubulin participates in the early stage of ICH pathogenesis.

Then we investigated whether HDAC6 inhibition by TubA or siRNA treatment had neuroprotective effects after ICH induction. Our results reflect that medium or high doses of TubA (25, 40 mg/kg) both improved neurological deficits and markedly inhibited cell apoptosis in the perihematomal tissue at 3rd day post‐ICH. Meanwhile, we demonstrated that TubA or siRNA can also increase cell viability as well as inhibit apoptosis of Hemin‐induced HBMECs. These results demonstrated that genetic or pharmacologic inhibition of HDAC6 exhibited beneficial effects for early brain injury post‐ICH.

The primary causes of brain injury post‐ICH are impaired BBB integrity and subsequent increased vascular permeability.^30^, ^31^ BBB disruption can generate brain edema,^32^ hematoma expansion, intracranial hypertension, and even midline shift.^5^, ^33^ Previous researches have shown that the peak of cerebral edema occurs on the 3rd day after ICH^34^ and indirectly indicated that the integrity of BBB is fragile at this time point. Hence, the effect of HDAC6 inhibition on BBB disruption after ICH was assessed in subsequent cellular and animal experiment. For in vivo experiment, TubA markedly reduced BBB permeability and ipsilateral brain edema. For in vitro experiment, TubA or HDAC6 knockdown significantly reduced endothelial permeability by measurement of FITC‐dextran extravasation. Thus, we preliminarily confirmed that HDAC6 inhibition alleviates the breakdown of BBB after ICH.

BBB is composed of a monolayer of endothelial cells (ECs) structure and numerous cell–cell junctional complexes for maintaining the integrity of BBB. Cell–cell junctional complexes mainly including tight junctions (TJs) and adherens junctions (AJs). TJs are situated on the apical membrane of ECs and consist of transmembrane proteins and cytoplasmic proteins that connect transmembrane proteins with the cytoskeleton.^35^ Claudin‐5 and occludin are the main transmembrane molecules of tight junctions mediating endothelial cell integrity. Besides, occludin binds to the cytoskeleton via cytoplasmic proteins zonula occludens‐1(ZO‐1). Contact in AJs is established mainly through VE‐cadherin. They also interact with the cytoskeleton via cytoplasmic anchor proteins such as catenins.^31^, ^36^ Under physiological conditions, actin in resting endothelium exists in the form of monomeric globular actin (G‐actin). Once activated, globular actin aggregates into filamentous actin (F‐actin), and this transition induces contractile stress fibers formation.^37^ Subsequently, part of the TJ proteins demounted at cell–cell contact and internalized due to the tension transmission,^38^, ^39^ thus expanding the endothelial gaps and increasing the BBB permeability. Thereby, inhibition of stress fiber formation in endothelial cells, which can promote the stability of cytoskeleton, represents a reasonable treatment strategy for blood–brain barrier protection after ICH.^32^

In our study, we detected the levels of TJ proteins (ZO‐1 and occludin) by western blot and observed the continuity of TJs by TEM to assess microvascular integrity. Previous study has demonstrated that HDACs inhibitors (valproic acid, Vorinostat and sodium butyrate) could up‐regulate the specific TJs proteins and this regulation was dependent on protein kinase activity.^40^ As expected, HDAC6 inhibition by TubA rescued the degradation of ZO‐1 and occludin as well as repaired TJs disruption after ICH. Besides, we demonstrated TubA and HDAC6 siRNA reduced stress fibers formation in cellular and animal model of ICH. These evidences suggested that selective HDAC6 inhibition prevented the BBB broke down via inhibition the formation of stress fiber and upregulation of TJ proteins. However, detailed molecular mechanisms of these effects observed by HDAC6 inhibition in ICH models have yet to be determined.

Acetylation and deacetylation of cell–cell junction and cytoskeleton proteins may serve as important mechanisms for controlling the dynamics of endothelial barrier integrity.^41^, ^42^ Indeed, the filaments of F‐actin are not immediately involved in progression of endothelial barrier dysfunction, but microtubules are the structure responsible for initial stages of this process.^43^, ^44^ Disassembly of microtubules promotes the reorganization of cortical actin rim into stress fibers as well as results in formation of intercellular gaps.^45^, ^46^, ^47^, ^48^ Furthermore, the acetylation α‐tubulin (at Lys40) was responsible for stabilizing microtubule structures.^49^, ^50^ Previous study found that TubA treatment can inhibit TNF‐induced microtubule disassembly and subsequent actin stress fiber formation in endothelial cells by preventing deacetylation of α‐tubulin.^20^ In consistent with these observations, our results showed that HDAC6 inhibition increased the acetylation of substrate α‐tubulin and promoted the stability of cytoskeleton in vivo and in vitro model of ICH. Taken all into consideration, we suggested that the effects of HDAC6 inhibition on cytoskeleton may be exerted via upregulating the acetylated α‐tubulin and subsequently stabilizing the endothelial microtubule structures.

## 5 CONCLUSIONS

Our research suggested that HDAC6 contributes, or at least partly, to the early pathophysiological process of brain injury after ICH. Besides, pharmacological inhibition or genetic inhibition of HDAC6 exerted protective effects on BBB. Furthermore, the neuroprotective effects were exhibited via upregulating the acetylated α‐tubulin and inhibiting stress fiber formation. In all, HDAC6 may be a promising target to fight against ICH‐induced BBB disruption.

## AUTHOR CONTRIBUTIONS

Concept and design: Chunli Chen and Cuiying Peng. Data collection and analysis: Cuiying Peng and Yilin Wang. Drafting of the article: Cuiying Peng and Chunli Chen. Critical revision of the article for important intellectual content: Yilin Wang and Zhiping Hu. All the authors approved the final article.

## FUNDING INFORMATION

This work was supported by National Natural Science Foundation of China (Grant no. 81974212 and 82101542), Natural Science Foundation of Hunan province (Grant no. 2021JJ40821), and Scientific Research Launch Project for new employees of the Second Xiangya Hospital of Central South University.

## CONFLICT OF INTEREST STATEMENT

The authors have no conflicts of interests.

## Supporting information


Table S1.

Table S2.

Appendix S1.

## Data Availability

The data that support the findings of this study are available from the corresponding author upon reasonable request.

## References

[1] van Asch CJ , Luitse MJ , Rinkel GJ , van der Tweel I , Algra A , Klijn CJ . Incidence, case fatality, and functional outcome of intracerebral haemorrhage overtime, according to age, sex, and ethnic origin: a systematic review and meta‐analysis. Lancet Neurol. 2010;9(2):167‐176. doi:10.1016/s1474-4422(09)70340-0 20056489

[2] Xi GH , Keep RF , Hoff JT . Mechanisms of brain injury after intracerebral haemorrhage. Lancet Neurol. 2006;5(1):53‐63. doi:10.1016/s1474-4422(05)70283-0 16361023

[3] Hemphill JC , Greenberg SM , Anderson CS , et al. Guidelines for the management of spontaneous intracerebral hemorrhage: a guideline for healthcare professionals from the American Heart Association/American Stroke Association. Stroke. 2015;46(7):2032‐2060. doi:10.1161/str.0000000000000069 26022637

[4] Chen S , Yang Q , Chen G , Zhang JH . An update on inflammation in the acute phase of intracerebral hemorrhage. Transl Stroke Res. 2015;6(1):4‐8. doi:10.1007/s12975-014-0384-4 25533878

[5] Selim M , Sheth KN . Perihematoma edema: a potential translational target in intracerebral hemorrhage? Transl Stroke Res. 2015;6(2):104‐106. doi:10.1007/s12975-015-0389-7 25693976 PMC4359064

[6] Keep RF , Zhou N , Xiang J , Andjelkovic AV , Hua Y , Xi G . Vascular disruption and blood‐brain barrier dysfunction in intracerebral hemorrhage. Fluids Barriers CNS. 2014;11:18. doi:10.1186/2045-8118-11-18 25120903 PMC4130123

[7] Chuang DM , Leng Y , Marinova Z , Kim HJ , Chiu CT . Multiple roles of HDAC inhibition in neurodegenerative conditions. Trends Neurosci. 2009;32(11):591‐601. doi:10.1016/j.tins.2009.06.002 19775759 PMC2771446

[8] Wang ZF , Fessler EB , Chuang DM . Beneficial effects of mood stabilizers lithium, valproate and lamotrigine in experimental stroke models. Acta Pharmacol Sin. 2011;32(12):1433‐1445. doi:10.1038/aps.2011.140 22056617 PMC4010202

[9] Bonsack F , Sukumari‐Ramesh S . Entinostat improves acute neurological outcomes and attenuates hematoma volume after intracerebral hemorrhage. Brain Res. 2021;1752:147222. doi:10.1016/j.brainres.2020.147222 33358731 PMC7903810

[10] Han Y , Chen L , Liu J , et al. A class I HDAC inhibitor rescues synaptic damage and neuron loss in APP‐transfected cells and APP/PS1 mice through the GRIP1/AMPA pathway. Molecules. 2022;27(13):4160. doi:10.3390/molecules27134160 35807406 PMC9268711

[11] Sukumari‐Ramesh S , Alleyne CH Jr , Dhandapani KM . The histone deacetylase inhibitor suberoylanilide hydroxamic acid (SAHA) confers acute neuroprotection after intracerebral hemorrhage in mice. Transl Stroke Res. 2016;7(2):141‐148. doi:10.1007/s12975-015-0421-y 26338677

[12] Hubbert C , Guardiola A , Shao R , et al. HDAC6 is a microtubule‐associated deacetylase. Nature. 2002;417(6887):455‐458. doi:10.1038/417455a 12024216

[13] Butler KV , Kalin J , Brochier C , Vistoli G , Langley B , Kozikowski AP . Rational design and simple chemistry yield a superior, neuroprotective HDAC6 inhibitor, tubastatin A. J Am Chem Soc. 2010;132(31):10842‐10846. doi:10.1021/ja102758v 20614936 PMC2916045

[14] Wang Z , Leng Y , Wang J , et al. Tubastatin A, an HDAC6 inhibitor, alleviates stroke‐induced brain infarction and functional deficits: potential roles of α‐tubulin acetylation and FGF‐21 up‐regulation. Sci Rep. 2016;6:19626. doi:10.1038/srep19626 26790818 PMC4726180

[15] Zhang J , Tan J , Hu Z , Chen C , Zeng L . HDAC6 inhibition protects against OGDR‐induced Golgi fragmentation and apoptosis. Oxid Med Cell Longev. 2019;2019:6507537. doi:10.1155/2019/6507537 31354911 PMC6636507

[16] Dompierre JP , Godin JD , Charrin BC , et al. Histone deacetylase 6 inhibition compensates for the transport deficit in Huntington's disease by increasing tubulin acetylation. J Neurosci. 2007;27(13):3571‐3583. doi:10.1523/jneurosci.0037-07.2007 17392473 PMC6672116

[17] Zhang L , Liu C , Wu J , et al. Tubastatin a/ACY‐1215 improves cognition in Alzheimer's disease transgenic mice. J Alzheimers Dis. 2014;41(4):1193‐1205. doi:10.3233/jad-140066 24844691

[18] Wang M , Zhou C , Yu L , et al. Upregulation of MDH1 acetylation by HDAC6 inhibition protects against oxidative stress‐derived neuronal apoptosis following intracerebral hemorrhage. Cell Mol Life Sci. 2022;79(7):356. doi:10.1007/s00018-022-04341-y 35678904 PMC11073123

[19] Zhu Y , Zheng H , Chen C . Protective effects of histone deacetylase 6 specific inhibitor tubastatin a on subarachnoid hemorrhage in rats and the underlying mechanisms. Zhong Nan Da Xue Xue Bao Yi Xue Ban. 2023;48(2):172‐181. doi:10.11817/j.issn.1672-7347.2023.220167 36999463 PMC10930345

[20] Yu J , Ma Z , Shetty S , Ma M , Fu J . Selective HDAC6 inhibition prevents TNF‐α‐induced lung endothelial cell barrier disruption and endotoxin‐induced pulmonary edema. Am J Physiol Lung Cell Mol Physiol. 2016;311(1):L39‐L47. doi:10.1152/ajplung.00051.2016 27190059

[21] Rosenberg GA , Mun‐Bryce S , Wesley M , Kornfeld M . Collagenase‐induced intracerebral hemorrhage in rats. Stroke. 1990;21(5):801‐807. doi:10.1161/01.str.21.5.801 2160142

[22] Zeng J , Chen Y , Ding R , et al. Isoliquiritigenin alleviates early brain injury after experimental intracerebral hemorrhage via suppressing ROS‐ and/or NF‐κB‐mediated NLRP3 inflammasome activation by promoting Nrf2 antioxidant pathway. J Neuroinflamm. 2017;14(1):119. doi:10.1186/s12974-017-0895-5 PMC5470182 28610608

[23] Garcia JH , Wagner S , Liu KF , Hu XJ . Neurological deficit and extent of neuronal necrosis attributable to middle cerebral artery occlusion in rats. Statistical validation. Stroke. 1995;26(4):627‐634; discussion 635. doi:10.1161/01.str.26.4.627 7709410

[24] Marks PA , Miller T , Richon VM . Histone deacetylases. Curr Opin Pharmacol. 2003;3(4):344‐351. doi:10.1016/s1471-4892(03)00084-5 12901942

[25] Kawaguchi Y , Kovacs JJ , McLaurin A , Vance JM , Ito A , Yao TP . The deacetylase HDAC6 regulates aggresome formation and cell viability in response to misfolded protein stress. Cell. 2003;115(6):727‐738. doi:10.1016/s0092-8674(03)00939-5 14675537

[26] d'Ydewalle C , Krishnan J , Chiheb DM , et al. HDAC6 inhibitors reverse axonal loss in a mouse model of mutant HSPB1‐induced Charcot‐Marie‐tooth disease. Nat Med. 2011;17(8):968‐974. doi:10.1038/nm.2396 21785432

[27] Yang Y , Zhang K , Zhong J , et al. Stably maintained microtubules protect dopamine neurons and alleviate depression‐like behavior after intracerebral hemorrhage. Sci Rep. 2018;8(1):12647. doi:10.1038/s41598-018-31056-7 30140021 PMC6107628

[28] Yang Y , Zhang X , Ge H , et al. Epothilone B benefits nigrostriatal pathway recovery by promoting microtubule stabilization after intracerebral hemorrhage. J Am Heart Assoc. 2018;7(2):e007626. doi:10.1161/jaha.117.007626 29348323 PMC5850167

[29] Yang Y , Chen X , Feng Z , et al. MEC17‐induced α‐tubulin acetylation restores mitochondrial transport function and alleviates axonal injury after intracerebral hemorrhage in mice. J Neurochem. 2022;160(1):51‐63. doi:10.1111/jnc.15493 34407220

[30] Tschoe C , Bushnell CD , Duncan PW , Alexander‐Miller MA , Wolfe SQ . Neuroinflammation after intracerebral hemorrhage and potential therapeutic targets. J Stroke. 2020;22(1):29‐46. doi:10.5853/jos.2019.02236 32027790 PMC7005353

[31] Yu QJ , Tao H , Wang X , Li MC . Targeting brain microvascular endothelial cells: a therapeutic approach to neuroprotection against stroke. Neural Regen Res. 2015;10(11):1882‐1891. doi:10.4103/1673-5374.170324 26807131 PMC4705808

[32] Manaenko A , Yang P , Nowrangi D , et al. Inhibition of stress fiber formation preserves blood‐brain barrier after intracerebral hemorrhage in mice. J Cereb Blood Flow Metab. 2018;38(1):87‐102. doi:10.1177/0271678x16679169 27864464 PMC5757435

[33] Xi G , Keep RF , Hoff JT . Pathophysiology of brain edema formation. Neurosurg Clin N Am. 2002;13(3):371‐383. doi:10.1016/s1042-3680(02)00007-4 12486926

[34] Zhao H , Pan P , Yang Y , et al. Endogenous hydrogen sulphide attenuates NLRP3 inflammasome‐mediated neuroinflammation by suppressing the P2X7 receptor after intracerebral haemorrhage in rats. J Neuroinflammation. 2017;14(1):163. doi:10.1186/s12974-017-0940-4 28821266 PMC5563049

[35] Abbott NJ , Patabendige AA , Dolman DE , Yusof SR , Begley DJ . Structure and function of the blood‐brain barrier. Neurobiol Dis. 2010;37(1):13‐25. doi:10.1016/j.nbd.2009.07.030 19664713

[36] Gavard J , Gutkind JS . VE‐cadherin and claudin‐5: it takes two to tango. Nat Cell Biol. 2008;10(8):883‐885. doi:10.1038/ncb0808-883 18670447 PMC2666287

[37] Shi Y , Zhang L , Pu H , et al. Rapid endothelial cytoskeletal reorganization enables early blood‐brain barrier disruption and long‐term ischaemic reperfusion brain injury. Nat Commun. 2016;7:10523. doi:10.1038/ncomms10523 26813496 PMC4737895

[38] Stamatovic SM , Keep RF , Wang MM , Jankovic I , Andjelkovic AV . Caveolae‐mediated internalization of occludin and claudin‐5 during CCL2‐induced tight junction remodeling in brain endothelial cells. J Biol Chem. 2009;284(28):19053‐19066. doi:10.1074/jbc.M109.000521 19423710 PMC2707189

[39] Vandenbroucke E , Mehta D , Minshall R , Malik AB . Regulation of endothelial junctional permeability. Ann N Y Acad Sci. 2008;1123:134‐145. doi:10.1196/annals.1420.016 18375586

[40] Bordin M , D'Atri F , Guillemot L , Citi S . Histone deacetylase inhibitors up‐regulate the expression of tight junction proteins. Mol Cancer Res. 2004;2(12):692‐701. 15634758

[41] Saito S , Lasky JA , Guo W , et al. Pharmacological inhibition of HDAC6 attenuates endothelial barrier dysfunction induced by thrombin. Biochem Biophys Res Commun. 2011;408(4):630‐634. doi:10.1016/j.bbrc.2011.04.075 21531207 PMC3100403

[42] Fang Z , Wang X , Sun X , Hu W , Miao QR . The role of histone protein acetylation in regulating endothelial function. Front Cell Dev Biol. 2021;9:672447. doi:10.3389/fcell.2021.672447 33996829 PMC8113824

[43] Alieva IB , Zemskov EA , Smurova KM , Kaverina IN , Verin AD . The leading role of microtubules in endothelial barrier dysfunction: disassembly of peripheral microtubules leaves behind the cytoskeletal reorganization. J Cell Biochem. 2013;114(10):2258‐2272. doi:10.1002/jcb.24575 23606375 PMC3901434

[44] Smurova KM , Verin AD , Alieva IB . The effect of rho‐kinase inhibition depends on the nature of factors that modify endothelial permeability. Tsitologiia. 2011;53(4):359‐366. 21675216

[45] Alieva IB . Role of microtubule cytoskeleton in regulation of endothelial barrier function. Biochemistry. 2014;79(9):964‐975. doi:10.1134/s0006297914090119 25385022

[46] Bogatcheva NV , Verin AD . The role of cytoskeleton in the regulation of vascular endothelial barrier function. Microvasc Res. 2008;76(3):202‐207. doi:10.1016/j.mvr.2008.06.003 18657550 PMC2586393

[47] Prasain N , Stevens T . The Actin cytoskeleton in endothelial cell phenotypes. Microvasc Res. 2009;77(1):53‐63. doi:10.1016/j.mvr.2008.09.012 19028505 PMC2700738

[48] Shivanna M , Srinivas SP . Microtubule stabilization opposes the (TNF‐alpha)‐induced loss in the barrier integrity of corneal endothelium. Exp Eye Res. 2009;89(6):950‐959. doi:10.1016/j.exer.2009.08.004 19695246 PMC2784278

[49] Robson SJ , Burgoyne RD . Differential localisation of tyrosinated, detyrosinated, and acetylated alpha‐tubulins in neurites and growth cones of dorsal root ganglion neurons. Cell Motil Cytoskeleton. 1989;12(4):273‐282. doi:10.1002/cm.970120408 2655938

[50] Webster DR , Borisy GG . Microtubules are acetylated in domains that turn over slowly. J Cell Sci. 1989;92(Pt 1):57‐65. doi:10.1242/jcs.92.1.57 2674164

