# Supplementary material for: Selective HDAC6 inhibition protects against blood–brain barrier dysfunction after intracerebral hemorrhage
Source: CNS Neurosci Ther. 2023 Sep 4;30(3):e14429. doi: 10.1111/cns.14429 (PMC10915991; doi:10.1111/cns.14429)
Supplement: Supplementary file 1 — Table S1. [file CNS-30-e14429-s003.docx]

**Supplementary Material 1**

**Table S1 Rats Assignment and Use**

| **Group** | **Garcia** | **WB** | **IF** | **BWC** | **TUNEL** | **EB** | **TEM** | **Rats**  **Used** | **Rats**  **Died** | **Rats**  **in Total** | **Mortality**  **Rate** |
| --- | --- | --- | --- | --- | --- | --- | --- | --- | --- | --- | --- |
| **Experiment 1.**  **Time-course** |  |  |  |  |  |  |  |  |  |  |  |
| **Sham 6h** |  | 3 |  |  |  |  |  | **3** | 0 | **3** | 0% |
| **Sham 1d** |  | 3 |  |  |  |  |  | **3** | 0 | **3** | 0% |
| **Sham 2d** |  | 3 |  |  |  |  |  | **3** | 0 | **3** | 0% |
| **Sham 3d** |  | 3 |  |  |  |  |  | **3** | 0 | **3** | 0% |
| **Sham 7d** |  | 3 |  |  |  |  |  | **3** | 0 | **3** | 0% |
| **NO. of Sham** |  | **15** |  |  |  |  |  | **15** | **0** | **15** |  |
|  |  |  |  |  |  |  |  |  |  |  |  |
| **ICH 6h** |  | 3 |  |  |  |  |  | **3** | 0 | **3** | 0% |
| **ICH 1d** |  | 3 |  |  |  |  |  | **3** | 1 | **4** | 25% |
| **ICH 2d** |  | 3 |  |  |  |  |  | **3** | 1 | **4** | 25% |
| **ICH 3d** |  | 3 | 3 |  |  |  |  | **6** | 3 | **9** | 33.3% |
| **ICH 7d** |  | 3 |  |  |  |  |  | **3** | 0 | **3** | 0% |
| **NO. of ICH** |  | **15** | **3** |  |  |  |  | **18** | **5** | **23** | 21.7% |
|  |  |  |  |  |  |  |  |  |  |  |  |
| **Experiment 2.**  **Neuroprotective effect of TubA** |  |  |  |  |  |  |  |  |  |  |  |
| **Sham** | 12 |  |  |  | 3 |  |  | **15** | 0 | **15** | 0% |
| **ICH+Vehicle** | 12 |  |  |  | 3 |  |  | **15** | 5 | **20** | 25% |
| **ICH****+TubA 25mg/kg** | 12 |  |  |  | 3 |  |  | **15** | 3 | **18** | 16.7% |
| **ICH+TubA 40mg/kg** | 12 |  |  |  | 3 |  |  | **15** | 2 | **17** | 11.8% |
| **NO. of Rats** | **48** |  |  |  | **12** |  |  | **60** | **10** | **70** | 14.3% |
|  |  |  |  |  |  |  |  |  |  |  |  |
| **Experiment 3.** |  |  |  |  |  |  |  |  |  |  |  |
| **Effect of TubA on BBB** |  |  |  |  |  |  |  |  |  |  |  |
| **Sham** |  |  |  | 10 |  | 6 |  | **16** | 0 | **16** | 0% |
| **ICH+Vehicle** |  |  |  | 10 |  | 6 |  | **16** | 4 | **20** | 20% |
| **ICH+TubA 25mg/kg** |  |  |  | 10 |  | 6 |  | **16** | 2 | **18** | 11.1% |
| **ICH+TubA 40mg/kg** |  |  |  | 10 |  | 6 |  | **16** | 1 | **17** | 5.9% |
| **NO. of Rats** |  |  |  | **40** |  | **24** |  | **64** | **7** | **71** | 9.9% |
|  |  |  |  |  |  |  |  |  |  |  |  |
| **Experiment 4.**  **Mechanism** |  |  |  |  |  |  |  |  |  |  |  |
| **Sham^(1)^** |  | 3 | 3 |  |  |  | 3 | **9** | 0 | **9** | 0% |
| **ICH+Vehicle ^(2)^** |  | 3 | 3 |  |  |  | 3 | **9** | 2 | **11** | 18.2% |
| **ICH+TubA 25mg/kg** |  | 3 | 3 |  |  |  | 3 | **9** | 1 | **10** | 10% |
| **ICH+TubA 40mg/kg** |  | 3 | 3 |  |  |  | 3 | **9** | 0 | **9** | 0% |
| **NO. of Rats** |  | **12** | **12** |  |  |  | **12** | **36** | **3** | **39** | 7.7% |
| **In total** | **48** | **42** | **15** | **40** | **12** | **24** | **12** | **193** | **25** | **218** | **11.5%** |

**Garcia: Garcia test WB: western blot IF: immunofluorescence staining BWC: brain water content**

**TUNEL: TdT-mediated dUTP-biotin nick end labeling staining EB: Evans blue staining TEM: Electron microscope**
