# Supplementary material for: Selective HDAC6 inhibition protects against blood–brain barrier dysfunction after intracerebral hemorrhage
Source: CNS Neurosci Ther. 2023 Sep 4;30(3):e14429. doi: 10.1111/cns.14429 (PMC10915991; doi:10.1111/cns.14429)
Supplement: Supplementary file 2 — Table S2. [file CNS-30-e14429-s001.docx]

**Supplementary Material 2**

**Table S2 HDAC6 siRNA** **sequences.**

| **HDAC6 siRNA target** | **Sequence** |
| --- | --- |
| **target 1** | **CTTCGAAGCGAAATATTAAAA** |
| **target 2** | **GCAGTTAAATGAATTCCATTG** |
| **target 3** | **GAAACAACCCAGTACATGAAT** |

**The interference efficiency of three different siRNAs was tested, and we used target 3 in this study.**

**
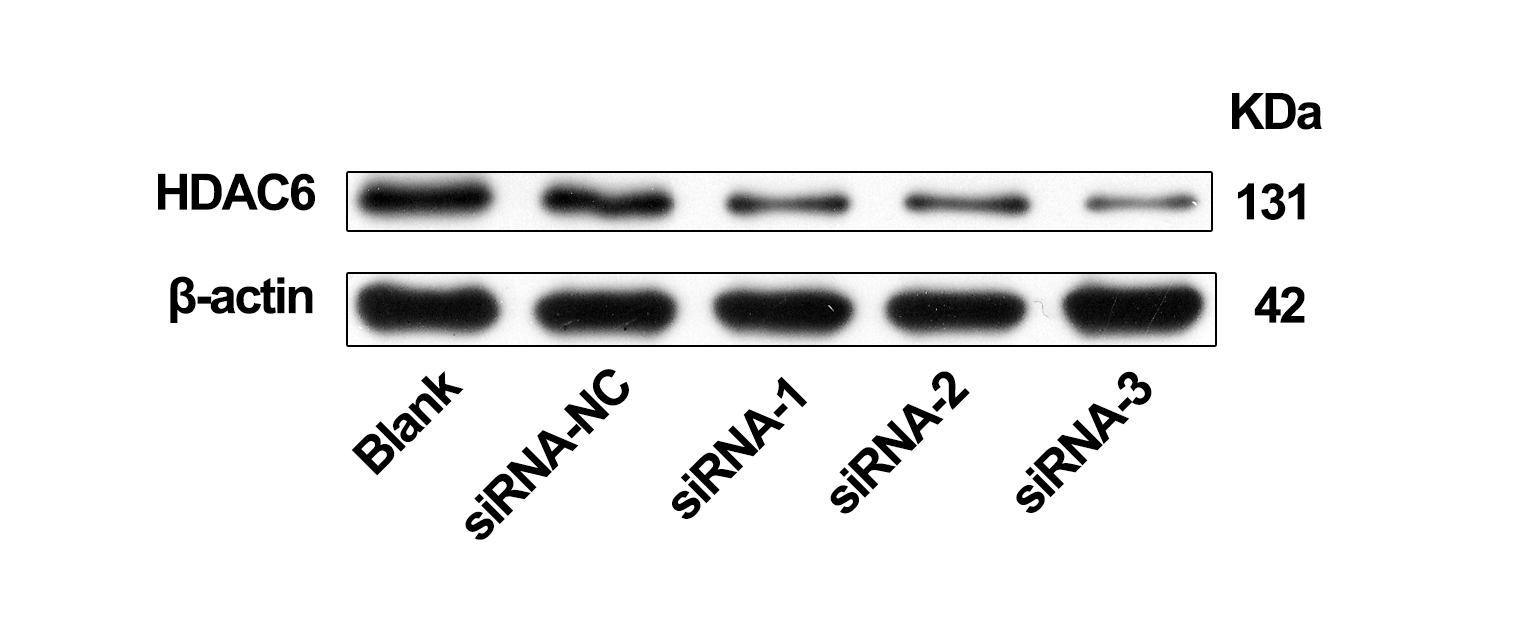
**
