# Supplementary figures and images for: Selective HDAC6 inhibition protects against blood–brain barrier dysfunction after intracerebral hemorrhage
Source: CNS Neurosci Ther. 2023 Sep 4;30(3):e14429. doi: 10.1111/cns.14429 (PMC10915991; doi:10.1111/cns.14429)

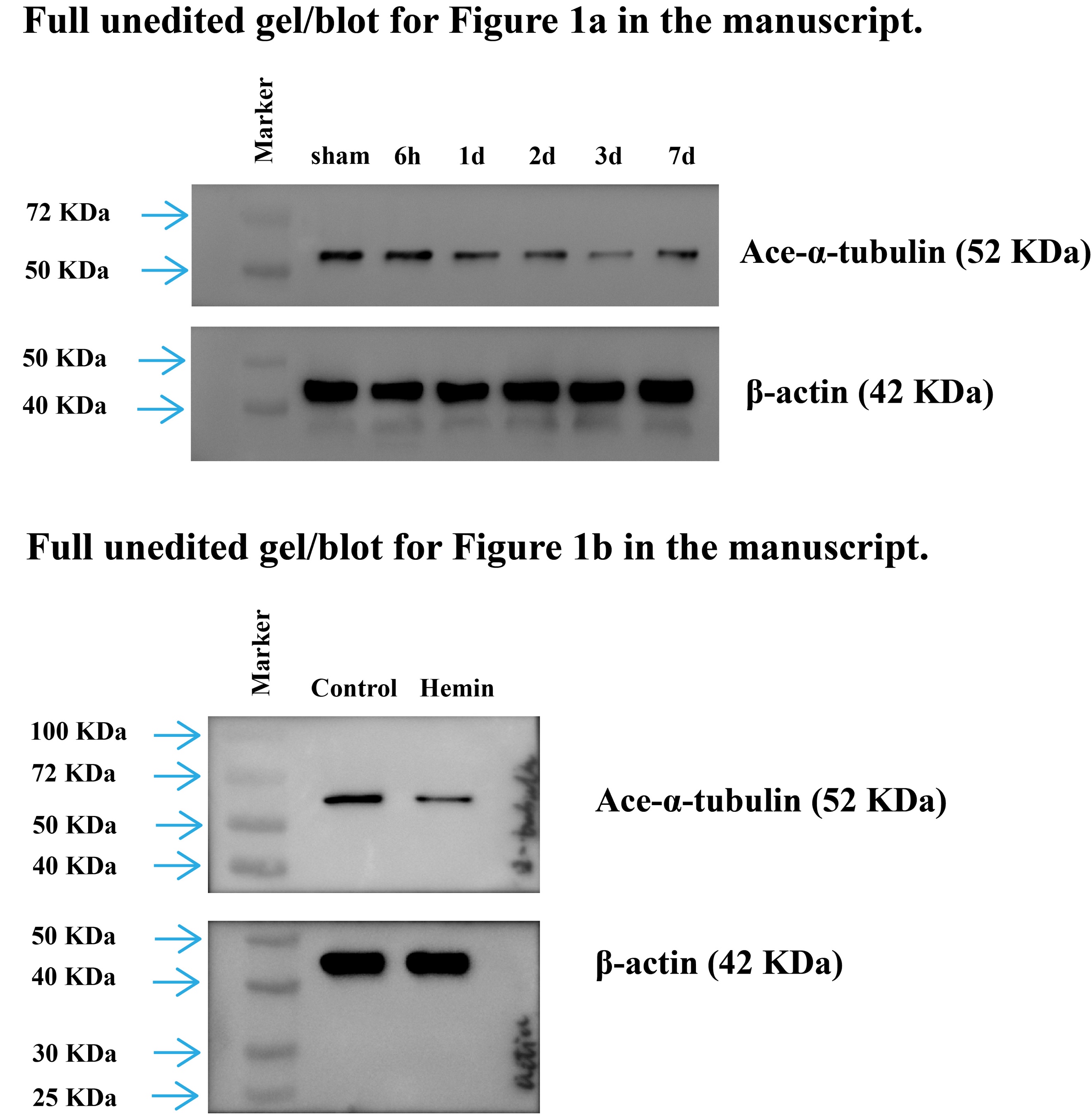





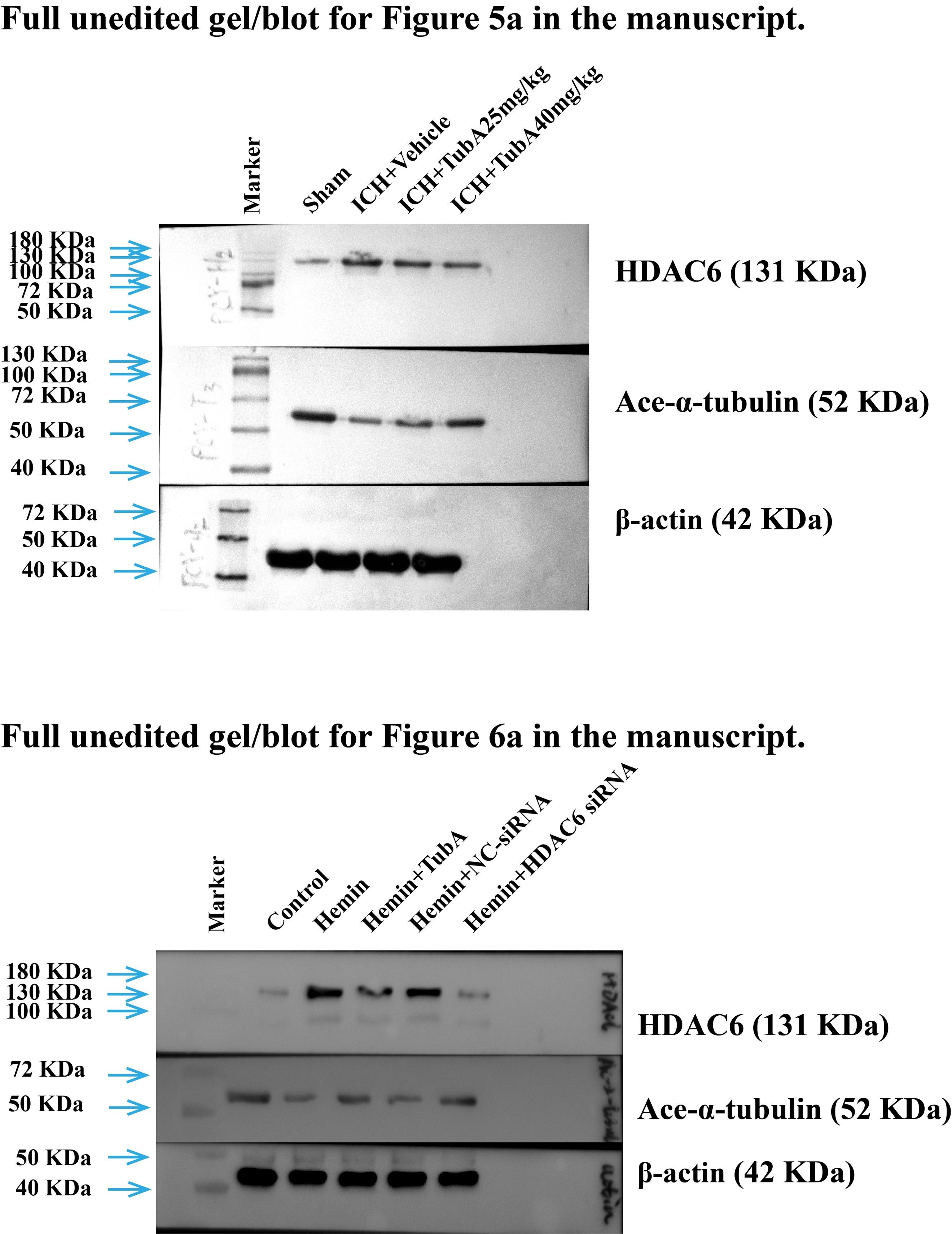

Supplement: Supplementary file 3 — Appendix S1. [file CNS-30-e14429-s002.docx]
